# Detection and Differentiation of Breast Cancer Sub-Types using a cPLA2α Activatable Fluorophore

**DOI:** 10.1038/s41598-019-41626-y

**Published:** 2019-04-16

**Authors:** Michael G. Chiorazzo, Hanna Maja Tunset, Anatoliy V. Popov, Berit Johansen, Siver Moestue, E. James Delikatny

**Affiliations:** 1 Department of Radiology, Perelman School of Medicine, University of Pennsylvania, Philadelphia, Pennsylvania 19104 United States; 2 Department of Circulation and Medical Imaging, Norwegian University of Science and Technology, Trondheim, 7491 Norway; 3 Department of Biology, Norwegian University of Science and Technology, Trondheim, 7491 Norway; 4 Avexxin AS, Department of Biology, Norwegian University of Science and Technology, N-7491 Trondheim, Norway; 5 Department of Laboratory Medicine, Children’s and Women’s Health, NTNU, The Norwegian University of Science and Technology, Trondheim, 7489 Norway

## Abstract

Cytosolic phospholipase A2α (cPLA2α) has been shown to be elevated in breast cancer and is a potential biomarker in the differentiation of molecular sub-types. Using a cPLA2α activatable fluorophore, DDAO arachidonate, we explore its ability to function as a contrast agent in fluorescence-guided surgery. In cell lines ranging in cPLA2α expression and representing varying breast cancer sub-types, we show DDAO arachidonate activates with a high correlation to cPLA2α expression level. Using a control probe, DDAO palmitate, in addition to cPLA2α inhibition and genetic knockdown, we show that this activation is a result of cPLA2α activity. In mouse models, using an *ex vivo* tumor painting technique, we show that DDAO arachidonate activates to a high degree in basal-like versus luminal-like breast tumors and healthy mammary tissue. Finally, we show that using an *in vivo* model, orthotopic basal-like tumors give significantly high probe activation compared to healthy mammary fat pads and surrounding tissue. Together we conclude that cPLA2α activatable fluorophores such as DDAO arachidonate may serve as a useful contrast agent for the visualization of tumor margins in the fluorescence-guided surgery of basal-like breast cancer.

## Introduction

Fluorescence guided surgery is an emerging technique that utilizes fluorescence contrast agents to aid in the identification of tumor margins during surgical resection^1–10^. Surgical resection of breast cancer remains the leading form of treatment and relapse free survival is directly correlated with complete removal of the primary tumor^11–13^. Fluorescent contrast agents, which enhance the ability to discern tumor margins, could therefore play a crucial role in improving disease treatment. In breast cancer specifically, there is a great a need for improved tumor resection as the positive surgical margin rates have been shown to range from 5–49% based on the study performed^14^.

Several fluorescent contrast agents have been explored for their potential clinical use as intraoperative agents including indocyanine green, fluorescein, and methylene blue^2,3,7,8,15^. These agents rely on the Enhanced Permeability and Retention (EPR) effect, a characteristic associated with disordered tumor vasculature which results in increased accumulation and retention of high molecular weight particles^16^. Optimization of dye dosage and plasma half-life is necessary in order to decrease background from systemic circulation while retaining high tumor signal^17^. Efforts to improve signal to noise have been made through the targeting of fluorophores to cancer signatures with varying success^17–22^. An alternative solution is the use of enzyme targeted activatable fluorophores that remain non-fluorescent until selectively acted upon^23–31^. A single enzyme, cleaving multiple substrates, can result in rapid signal amplification^32^. Unlike targeted fluorophores, activatable fluorophores provide a measure of enzyme activity that may differ from expression level. Additionally, activatable fluorophores could be applied to tissue in real-time during surgery, avoiding the need for systemic administration and potentially reducing off-target effects.

cPLA2α has been of increasing interest to the oncology field for its elevated activity in certain cancers^33–35^. In breast cancer, cPLA2α expression has been shown to be correlated with molecular sub-type, and is expressed at high levels in the basal-like, triple negative phenotype^36–38^. Using clinical mRNA expression data from a publically available online tool, it can be shown that basal-like patients with higher expression of cPLA2α have a significantly decreased rate of relapse free survival (p = 0.00024)^39^. In addition, cPLA2α inhibition has been shown to delay tumor growth in preclinical models^40^.

**Figure 1:**
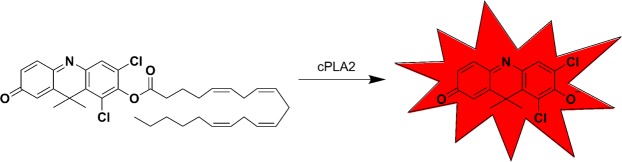
DDAO arachidonate structure and activation. DDAO arachidonate, consisting of DDAO esterified to arachidonic acid is non-fluorescent at 600 nm. Cleavage of the ester linkage by cPLA2α, releases free DDAO, which becomes fluorescent with an ex/em maxima of 600/660 nm.

Activatable fluorophores with selectivity towards cPLA2α have been described based on a caged fluorescence design^41^. DDAO arachidonate, consists of arachidonic acid esterified to the fluorophore DDAO (9*H*-(1,3-dichloro-9,9-dimethylacridin-2-one-7-yl, Fig. 1). Esterification of DDAO results in ablated fluorescence and serves as a caged mechanism of activation. cPLA2α has high selectivity towards arachidonic acid and therefore can cleave DDAO arachidonate but not DDAO palmitate, a control probe useful in the evaluation of non-specific probe cleavage. Following cleavage of esterified DDAO, fluorescence can be detected with a broad excitation between 600–650 nm and an emission maximum at 660 nm. This wavelength lies at the edge of the near infrared (NIR) window, a region of the electromagnetic spectrum with minimal absorption from biological tissue^17^. NIR fluorophores are therefore ideal for the development of contrast agents for fluorescence-guided surgery. Here, we explore the use of DDAO arachidonate to detect cPLA2α activity in several breast cancer sub-types both in cells and *in vivo* mouse models.

## Results

### Intracellular evaluation of cPLA2α activity by DDAO arachidonate

Three breast cancer cell lines were chosen to represent the three most well defined sub-types of breast cancer. MDA-MB-231 is a basal-like cell line representing triple-negative breast cancer. SKBR3 is a HER2-like cell line representing HER2 overexpressing breast cancer. MCF-7 is a luminal-like cell line representing estrogen receptor positive breast cancer. An additional cell line, 4175-Luc+, is a lung metastatic derivative of the MDA-MB-231 cell line engineered to overexpress luciferase and green fluorescent protein^42^.

**Figure 2:**
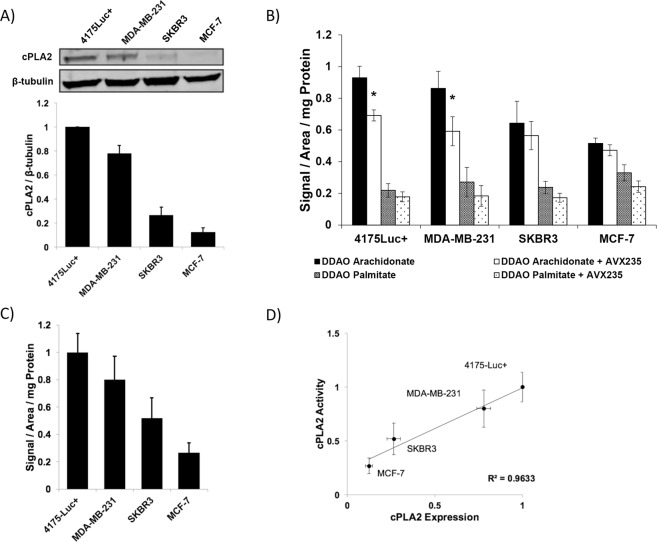
DDAO arachidonate activation in cell lines varying in cPLA2α expression. (**A**) cPLA2α protein expression in four breast cancer cell lines representing three different molecular sub-types: Basal-like triple negative (4175-Luc+ and MDA-MB-231), HER2-overexpressing (SKBR3), and luminal-like (MCF-7). Membrane was cut and lower portion was blotted for ß-tubulin as a loading control. Full gel is provided in Fig. S6. (**B**) Fluorescence (ex/em: 685/720 nm) in cells treated with DDAO arachidonate or DDAO palmitate. cPLA2α inhibition through AVX235 treatment resulted in significant fluorescence reduction in 4175-Luc+ and MDA-MB-231 cells (p = 0.006 and 0.03, respectively). (**C**) Adjusted cPLA2α activity derived from (**B**) through the subtraction of DDAO palmitate fluorescence from DDAO arachidonate fluorescence, plotted relative to 4175-Luc+ activity. (**D**) Correlation of cPLA2α expression (**A**) to cPLA2α activity (**C**) showed a strong positive correlation (R^2^ = 0.9633) between cPLA2α expression and activity.

Cell lines were analyzed by immunoblot to determine the relative protein levels of cPLA2α (Fig. 2A). Consistent with previous literature, it was found that MCF-7 cells display the lowest levels of cPLA2α, with SKBR3 cells having 2-fold higher expression and MDA-MB-231 cells having 4-fold higher expression^36^. 4175-Luc+ cells displayed the highest cPLA2α expression, even greater than their parent cell line, MDA-MB-231.

The cPLA2α activity within these established cell lines was evaluated using, the cPLA2α activatable fluorophore, DDAO arachidonate. Cells were cultured in 96 well, clear bottom, black walled plates. DDAO arachidonate was added to cells with and without 10 µM of the cPLA2α inhibitor AVX235 (Fig. S1). The probe DDAO palmitate, which is not sensitive to the actions of cPLA2α, was also utilized in separate wells as a control. Fluorescence was acquired after four hours in the 700 nm channel of the Odyssey imaging system (Fig. 2A). It was determined that 4175-Luc+ cells gave the highest increase in fluorescence, followed by the MDA-MB-231 cell line, then the SKBR3 cell line, then the MCF-7 cell line which had the lowest level of fluorescence generation (Fig. 2B). AVX235, a highly potent cPLA2α inhibitor, led to significant reduction of DDAO arachidonate fluorescence in 4175-Luc+ and MDA-MB-231 cells (p = 0.006 and p = 0.03, respectively). In contrast, DDAO arachidonate fluorescence was not significantly reduced by AVX235 in SKBR3 and MCF-7 cells (Fig. 2B). AVX235 had no effect on non-specific fluorescence generated by DDAO palmitate. The DDAO arachidonate fluorescence was adjusted by subtracting the non-selective DDAO palmitate fluorescence in order to quantify the relative cPLA2α activity within these cell lines (Fig. 2C). Correlation of cPLA2α protein expression with relative DDAO arachidonate signal resulted in a strong positive correlation (R^2^ = 0.9633) (Fig. 2D).

**Figure 3:**
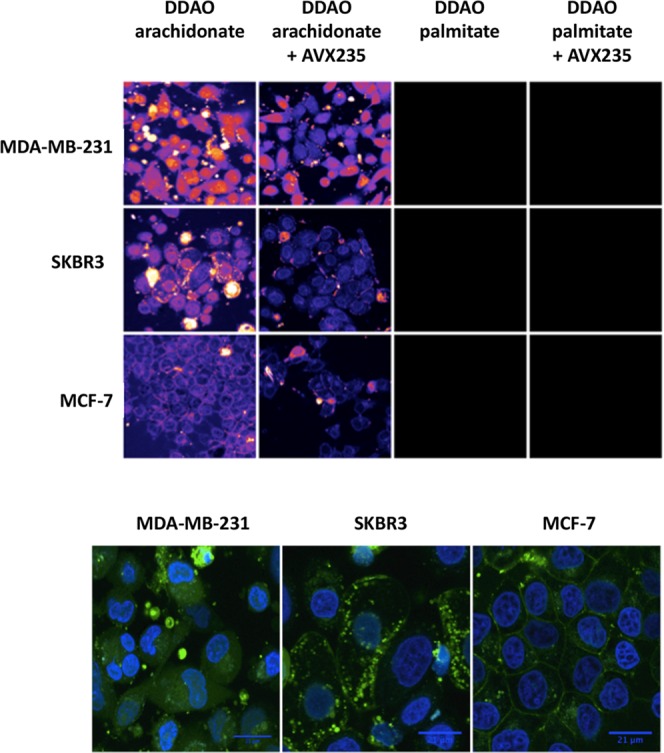
Confocal fluorescence microscopy of DDAO arachidonate and DDAO palmitate activation. (**A**) MDA-MB-231, SKBR3, and MCF7 cells treated with DDAO arachidonate or DDAO palmitate for four hours with and without cPLA2α inhibition. cPLA2α activated DDAO fluorescence is shown as a heat map with brighter areas representing higher fluorescence intensity. The highest fluorescence was observed in MDA-MB-231 cells, followed by SKBR3, then MCF-7. In all cases, cPLA2α inhibition appeared to decrease fluorescence. For all cell lines, DDAO palmitate resulted in considerably less fluorescence signal. (**B**) Subcellular distribution of DDAO arachidonate following four hours of probe incubation. DDAO fluorescence is shown in green, and nuclear stain (HOECHST 33342) is shown in blue. Fluorescence is not adjusted for relative intensity, but is optimized for visualization of probe distribution.

To determine whether the inability to significantly reduce fluorescence in MCF-7 and SKBR3 cells using AVX235 was the result of limitations in signal detection, we repeated our activation experiments using fluorescence confocal microscopy (Fig. 3A). Similar to the spectroscopic analysis, DDAO fluorescence was highest in basal-like breast cancer cell lines and lowest in luminal-like cell lines. Furthermore, a clear reduction in fluorescence was observed in all cell lines following incubation with AVX235. In addition to differing fluorescence intensity between cell lines, a clear difference in sub-cellular distribution of DDAO was observed (Fig. 3B). MDA-MB-231 cells showed DDAO fluorescence evenly dispersed throughout the cytoplasm. SKBR3 cells showed cytoplasmic activation, but the fluorescence was confined to small vesicles. MCF-7 cells showed low general activation, with some fluorescence at the plasma membrane. Further investigation of this effect with time was performed with wide-field fluorescence microscopy over the course of 70 minutes in 4175-Luc+ cells (Fig. S2). Here, fluorescence appeared at first concentrated in small vesicles near the perinuclear region. With time, the vesicles disappeared and the fluorescence was distributed evenly throughout the cytoplasm.

**Figure 4:**
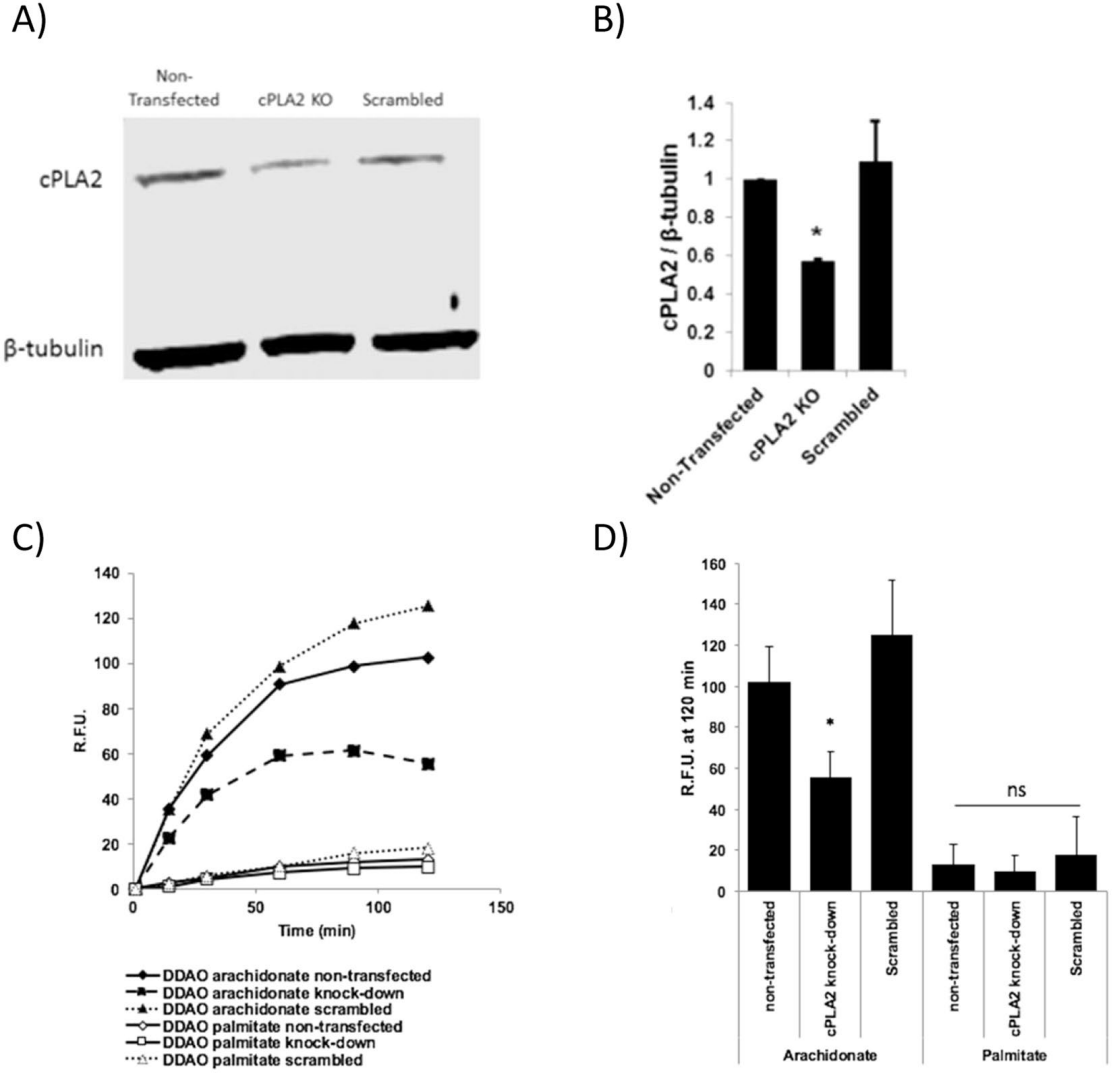
DDAO arachidonate fluorescence in cPLA2α knockdown 4175-Luc+ cells. (**A**) Protein expression in 4175-Luc+ cells, cPLA2α CRISPR knockout plasmid transfected cells, and scrambled CRISPR knockout plasmid transfected cells. Full gel is presented. (**B**) Quantitated cPLA2α protein levels, taken from (**A**), relative to beta-tubulin. Significant knockdown of cPLA2α protein was observed in cPLA2α CRISPR plasmid transfected cells (p = 8.2 × 10^−7^). (**C**) Relative fluorescence of DDAO arachidonate or DDAO palmitate with time in cPLA2α knockdown cell lines. (**D**) Quantitated relative fluorescence at 120 minutes, taken from (**C**). Significant reduction of fluorescence was observed in cPLA2α knockdown cells treated with DDAO arachidonate (p = 1.58 × 10^−6^). No significant changes in fluorescence were observed when treated with DDAO palmitate.

In order to further validate that the observed fluorescence signal was a result of cPLA2α activity, 4175-Luc+ cells were transfected with a cPLA2α targeted CRISPR knockout vector (Santa Cruz Biotechnology). Fluorescence cell sorting was used to isolate vector expressing cells (Fig. S3). Immunoblotting revealed a 40% decrease in cPLA2α protein expression in CRISPR treated cells (Fig. 4A,B). Although complete genetic knockout was not achieved, DDAO arachidonate activation was evaluated using fluorescence spectroscopy and a significant decrease in fluorescence intensity was observed in the cPLA2α knockdown cells (p = 8.2 × 10^−7^) (Fig. 4C,D).

### *Ex vivo* detection of cPLA2α activity in mouse tumor models

In order to evaluate the use of DDAO arachidonate for tumor detection, 4175-Luc+ cells were grown as tumor xenografts in mice. DDAO arachidonate or DDAO palmitate were injected *i*.*v*. and whole-body fluorescence was monitored with time. High background fluorescence from probe cleavage in the intestines was observed, making resolution of tumor signal challenging (Fig. S4A). This activation was not significantly different between cPLA2α and control probes and is therefore likely related to non-cPLA2α mediated cleavage, probably due to pancreatic phospholipases in the digestive tract (Fig. S4B). This activation was confirmed through *ex vivo* organ analysis showing high intestinal fluorescence including the stomach, small intestine, and large intestine (Fig. S4C,D). Despite this, comparison of non-digestive tract organs revealed DDAO arachidonate tumor fluorescence to be significantly higher than heart, lungs, kidney, and muscle (p = 0.02, 0.03, 0.04, 0.04, respectively) (Fig. S4E).

**Figure 5:**
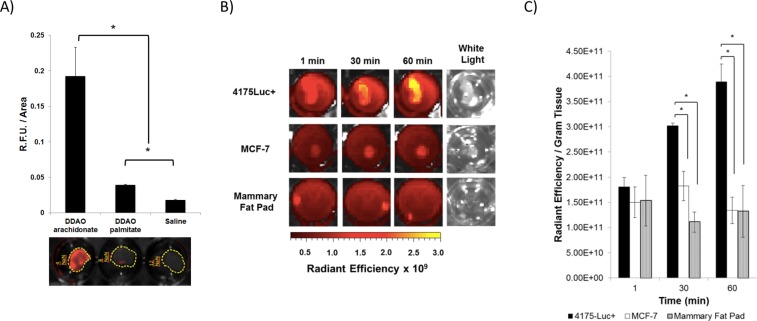
*Ex Vivo* Tumor Painting. (**A**) 4175-Luc+ orthotopic tumor slices incubated with DDAO arachidonate, DDAO palmitate, or saline for 3 hours followed by washing with PBS. Fluorescence was acquired in the LI-COR Pearl Impulse imaging system (700 nm channel). Significant higher DDAO arachidonate fluorescence was observed compared to DDAO palmitate and saline (p = 7 × 10^−6^ and 6.5 × 10^−6^ respectively). DDAO palmitate gave significantly higher fluorescence than saline control (p = 4.5 × 10^−4^). (**B**) 4175-Luc+ and MCF-7 orthotopic tumor slices and non-inoculated mammary fat pad slices were incubated with DDAO arachidonate. Fluorescence was acquired without washing over the course of 1 hour in the IVIS Spectrum imaging system (ex/em 640/680 nm). (**C**) Quantification of tissue fluorescence in (**B**). per gram of tissue. Significantly higher fluorescence was observed in 4175-Luc+ tumors versus MCF-7 and saline controls at 30 minutes (p = 0.002 and p = 0.04, respectively) and 1 hour (p = 0.001 and p = 0.04, respectively).

Activatable fluorophores offer the advantage of direct application to the tumor during surgery. Therefore, in order to avoid the background signal association with systemic delivery, the feasibility of applying DDAO arachidonate directly to 4175-Luc+ tumors was tested. Orthotopic tumors were excised from non-treated mice, sliced, and immediately placed in culture medium. Tumors were given 1 hour to adjust to *ex vivo* culture, then DDAO arachidonate, DDAO palmitate, or saline was applied to the tumor. After 3 hours, tumors were washed 2x with PBS to remove background signal and fluorescence was acquired on the LI-COR Pearl Imaging System (700 nm channel). Fluorescence was confirmed to be detectable on the LI-COR Pearl channel through imaging of free fluorophore at pH 7.4 and pH 5.0. At low pH the hydroxyl group in DDAO is protonated, which depletes fluorescence, mimicking the caged fluorescence seen with esterification (Fig. S5). In the painted tumors, significantly higher activation of DDAO arachidonate was observed compared to DDAO palmitate (p = 7 × 10^−6^) (Fig. 5A). In addition, the DDAO palmitate fluorescence was significantly higher than saline control (p = 4.5 × 10^−4^). *Ex vivo* fluorescence activation with time was compared between 4175-Luc+ tumors, MCF-7 tumors, and non-inoculated mammary fat pad (Fig. 5B). Significantly higher fluorescence activation was observed in 4175-Luc+ tumor slices when compared to MCF-7 tumor slices and non-inoculated mammary fat pad at 30 minutes (p = 0.002 and 0.04, respectively) and 1 hour (p = 0.001 and 0.04, respectively) (Fig. 5C).

### Application of DDAO arachidonate to assist in identification of tumor margins *in vivo*

**Figure 6:**
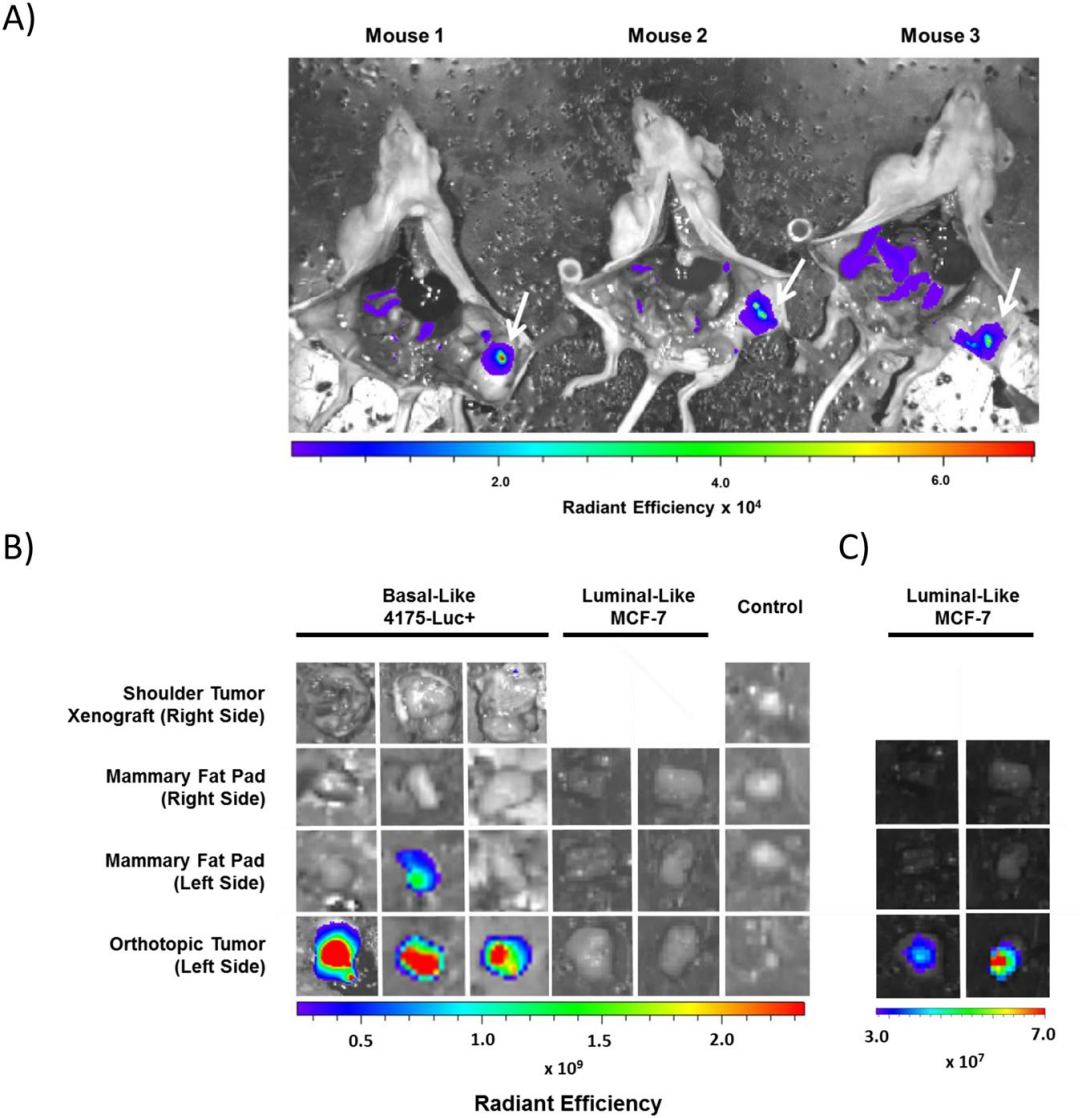
Intraoperative study of DDAO arachidonate. (**A**) DDAO arachidonate was injected i.p. directly under orthotopic tumors growing in the lower mammary fat pad adjacent to the peritoneal cavity. Following 1 hour, mice were euthanized, the peritoneal cavity was exposed and fluorescence was acquired (ex/em: 640/680 nm). Strong fluorescence signal occurred in areas overlapping the orthotopic tumor (indicated with a white arrow). (**B**) Orthotopic tumors, xenograft tumors, and mammary fat pads were dissected from mice, washed 1x with PBS and fluorescence was acquired (ex/em: 640/680). High fluorescence was detectable from 4175-Luc+ orthotopic tumors. (**C**) Signal from MCF-7 orthotopic tumors in (**B)** was low, but became detectable when detection sensitivity was increased, revealing tumor fluorescence higher than that of mammary fat pad.

In order to mimic painting of DDAO arachidonate in a surgical setting, 4175-Luc+ cells were grown as orthotopic tumors in the lower mammary fat pad directly below the peritoneal cavity. As an off-site control, cells were also grown as subcutaneous shoulder tumor xenografts, distal to the peritoneal cavity. DDAO arachidonate was then administered *i*.*p*. directly below the tumor. Following a one-hour incubation, mice were euthanized and the peritoneal cavity was exposed (Fig. 6A). High fluorescence was observed that overlapped with the tumor location (indicated with a white arrow). Minimal fluorescence activation was observed in the peritoneal cavity. Orthotopic tumor, mammary fat pads from opposing sides, and control shoulder tumor xenograft were excised, washed in PBS, and imaged for fluorescence (Fig. 6B). High fluorescence from orthotopic tumors was observed, which was significantly higher than fluorescence from the inoculated mammary fat pad, non-inoculated mammary fat pad, and the shoulder tumor xenograft (anatomically distal to site of injection) (p = 0.002, 0.001, 0.001, respectively, n = 5). The radiance measured in orthotopic tumors ranged from 7 to 12 fold higher than mammary fat pad signal. MCF-7 tumors (n = 2) subjected to identical evaluation showed significantly lower levels of fluorescence compared to 4175-Luc+ (n = 3, p = 0.01). However, adjusting for lower overall signal intensity in MCF-7 tumors revealed a significant activation of DDAO arachidonate in MCF-7 orthotopic tumors vs. non-inoculated and inoculated mammary fat pad (p = 0.009 and 0.01, respectively) (Fig. 6C).

## Discussion

cPLA2α has recently been shown to have an important role in breast cancer progression and to be a potential therapeutic target in basal-like breast cancer. cPLA2α expression levels have been shown to correlate with molecular sub-type, being highest in basal-like and lowest in luminal-like carcinoma^36,37^. cPLA2α is therefore is a powerful biomarker for the detection of aggressive, basal-like breast cancer. Using a newly developed, red fluorescent, cPLA2α targeted, activatable fluorophore (DDAO arachidonate) and control (DDAO palmitate) we sought to exploit this biomarker for the detection and differentiation of breast cancer in cultured cells and tumors.

To begin, we sought to assess whether activation of DDAO arachidonate varied between breast cancer sub-types expressing different levels of cPLA2α. Fluorescence spectroscopy indicated that activation was highest in basal-like triple negative cells, followed by HER2-like cells, and lowest in luminal-like cells. Fluorescence was highly correlated to enzyme expression level, indicating that cPLA2α activity is closely linked to expression. The absence of high signal from the DDAO palmitate probe indicates low non-selective activation. Use of a cPLA2α inhibitor, AVX235, resulted in significant reduction of fluorescence in basal-like cells but not HER2-like or luminal-like cells, although a trend of lowered signal was seen. It is possible that difficulties in signal detection were related to the inherently low cPLA2α activity, and detection sensitivity simply was not high enough to obtain statistical significance. Using confocal microscopy to examine fluorescence activation in individual cells appeared to confirm this hypothesis and a clear drop in cellular fluorescence was observed in all cell lines tested following incubation with AVX235. In addition to AVX235 inhibition experiments, CRISPR cPLA2α knockdown cells were generated to provide a more direct evaluation of the cPLA2α dependent activation of DDAO arachidonate. After treatment with a cPLA2α knockout plasmid, a 40% decrease in cPLA2α expression was observed, we refer to this cell line as cPLA2α knockdown. When analyzed for DDAO arachidonate activation, cPLA2α knockdown cells exhibited decreased fluorescence activation, which correlated to protein expression. This provides further evidence that the observed fluorescence signal is a direct result of cPLA2α activity. In all cell lines analyzed with confocal microscopy, several cells were observed with higher fluorescence intensity compared to surrounding cells. These cells had characteristic signs of stress including rounding up and detachment from the plate. It has previously been reported that cPLA2α signaling is active in apoptotic activation^43–46^. Therefore, DDAO arachidonate may have additional use in the evaluation of therapeutic response in cancer, or as a probe to guide the development of novel cPLA2α inhibitors.

Interestingly, the cell lines displayed differences in sub-cellular distribution of DDAO. Basal-like cells showed an even cytosolic distribution. SKBR3 cells showed signal contained to cytosolic vesicles. Finally, MCF-7 cells showed fluorescence distributed around the cellular membrane. Liposomes are taken up into cells through two main processes^47^. Membrane fusion between the liposome and cellular membrane can occur resulting in dispersion of the probe in the membrane. Alternatively, endocytosis of the liposomes followed by fusion or lipid exchange can occur^48^. Based on the extent of lipid exchange, transfer to lysosomes may occur and present a source for non-selective activation. It is possible that the cell lines display different uptake mechanisms resulting in different activation sites. Time course microscopy (Fig. S2) in 4175-Luc+ cells showed DDAO fluorescence appeared first in small cytosolic vesicles followed by dispersion of the signal throughout the cytoplasm. Therefore, it is also possible that the observed differences in subcellular distribution of fluorescence may be a function of time and may depend on the degree of cPLA2α activity.

An initial analysis of *in vivo* DDAO arachidonate activation was performed through systemic tail vein injection. This resulted in high background signal occurring most prominently in the intestines. DDAO arachidonate has also been shown to be susceptible to cleavage by pancreatic phospholipase (sPLA2 GIB)^41^. High intestinal fluorescence may therefore be attributed to sPLA2-mediated cleavage, leading to less native probe available for tumor tissue. Chemical modification of DDAO arachidonate to reduce sPLA2 activation might reduce this observed background. Furthermore, use of fluorophores similar to DDAO, with longer emission wavelengths, might improve detection following systemic administration^49^.

Due to the activatable nature of DDAO arachidonate, systemic administration may not be necessary. Instead topical application of probe to tissue in real-time during surgery could aid in the identification of tumor margins. In addition, DDAO arachidonate could potentially allow for rapid and sensitive detection of small cancer cell deposits in the form of ductal carcinoma *in situ* (DCIS) or in lymph nodes. In order to explore this potential use, we developed a technique for tumor painting, whereby non-treated tumors were excised, cultured and treated *ex vivo*. Tumor painting experiments showed, similar to cellular studies, that basal-like tumors activated DDAO arachidonate to a significantly higher degree compared to DDAO palmitate. The observed increase in signal from DDAO palmitate compared to saline indicates that there may be some contribution from non-cPLA2α mediated cleavage, either through other phospholipase A2 isoforms or non-specific hydrolysis. However, despite this, DDAO arachidonate showed a strong time-dependent activation that was significantly greater in basal-like tumors than luminal-like tumors.

In experiments designed to model fluorescence-guided surgical painting *in vivo*, we injected the probe *i*.*p*. close to the site of orthotopic tumor. We found that this approach substantially reduced background fluorescence from the intestine, producing strong signals localized to the region of the tumor. Distribution through this approach may be occurring through diffusion to nearby tissue or through lymphatic distribution. A previous comparison of routes for the administration of liposomes found that i.p. injected liposomes were absorbed through the lymphatic system^50^. Due to the heavy lymphatic nature of mammary tissue, it may be that the orthotopic tumors are receiving high doses of DDAO arachidonate before systemic distribution can occur. Regardless, the absence of signal from healthy mammary fat pads and surrounding tissue in the peritoneal cavity indicate activation through this route of administration occurs primarily in cancerous tissue. In one case, residual fluorescence was observed from the mammary fat pad in which the orthotopic tumor was grown. This may be the result of non-specific activation, inflammatory mediated processes, or the presence of residual tumor that was not entirely excised. The cause of this residual fluorescence was not explored, however we believe this highlights the potential utility of cPLA2α activity for tumor detection. Similar experiments with MCF-7 tumors indicated significant activation in tumors versus healthy mammary fat pad, albeit at levels significantly lower than basal-like tumors.

Taken together, DDAO arachidonate appears to be a useful research tool for the detection of cPLA2α activity in breast cancer cells lines. In addition, DDAO arachidonate may be useful for the fluorescence guided surgery of basal-like breast cancer in applications including the visualization of surgical margins. Topical application represents a feasible approach, with low background signal and high specificity that avoids the high intestinal activation that occurs with systemic administration. However, the anatomic location of the breast, combined with imaging systems that can isolate fluorescence to only breast cancer tissue may mitigate this effect in human patients^51–55^. In conclusion, DDAO arachidonate may be highly useful for enhancing detection of basal-like breast cancer.

## Methods

DDAO arachidonate and DDAO palmitate were synthesized as described previously^41^. HOECHST 333258 (Cat. # H1398) was generously provided by the Cellular and Molecular Imaging Core Facility at the Norwegian University of Science and Technology. The cPLA2α inhibitor, AVX235 (Fig. S1), was provided by Avexxin AS (Trondheim Norway)^56^. Egg phosphatidylcholine was purchased from Avanti Polar Lipids (Cat. # 840051C). MDA-MB-231, SKBR3, and MCF-7 cells were purchased from the American Type Culture Collection (ATCC). 4175-Luc+ cells were generously provided by Dr. Andy Minn^42^. Human cPLA2α monoclonal rabbit antibody (Cat. # 2832S) and human beta-tubulin rabbit monoclonal antibody (Cat. # 2128S) were purchased through Cell Signaling Technology. IRDye800 goat anti-rabbit secondary antibody (Cat. # 925–32211) was purchased through LI-COR Biosciences. Athymic NCR nu/nu 01B7 female mice were purchased through Charles River. CRISPR/Cas9 knockout (Cat. # sc-400678-KO-2) and control (Cat. # sc-418922) plasmids and UltraCruz reagent (Cat. # sc-395739) were purchased through Santa Cruz Biotechnology.

### Probe Formulation

DDAO arachidonate and DDAO palmitate were prepared in liposomes for all experiments. Probe was mixed with egg phosphatidylcholine in chloroform at a mole fraction of 0.05. The solvent was removed under a stream of nitrogen gas followed by high vacuum to remove trace chloroform. The dry lipid cake was suspended in sterile saline or 10 mM HEPES (pH 7.0) with 0.1 μM EDTA and 1.1 mM CaCl_2_. The final probe concentration was 100 µM for *in vitro* and 400 µM for *in vivo* assays. Liposomes were formed through sonication in a water bath sonicator until an optically clear suspension was obtained. Liposomes were used immediately following constitution.

### Cell Culture

All cell lines were cultured in Dulbecco’s Modified Eagle Medium (DMEM) supplemented with 10% fetal bovine serum (HyClone Laboratories), 1% L-glutamine (Mediatech), and 1% penicillin-streptomycin (Mediatech). 4175-Luc+ cells were maintained in 5 µg/mL blasticidin (Invitrogen, Cat. # ant-bl-05). For routine maintenance, cells were passaged at a ratio of 1:10 and used for up to 20 passages before being discarded. Following thawing, the cells were cultured for a minimum of one week before experiments. Mycoplasma evaluation was performed routinely using the MycoAlert Mycoplasma Detection Kit (Lonza Group, Cat. # LT07).

#### Cellular fluorescence activation assay

Cells were seeded in 96 well black-wall, clear bottom plates at 1.5–3.5 × 10^4^ cells per well, and incubated for 48 hours, optimized so that each cell line was ~80% confluent at the time of the experiment. Assays were initiated through addition of 10 µL of DDAO arachidonate or DDAO palmitate liposomes to 90 µL medium per well. Phenol red free medium was used for all optical experiments. For the cPLA2α inhibition experiments, cells were treated with 10 µM AVX235 for 2.5 hours prior to probe addition. Fluorescence was monitored on a LI-COR Odyssey CLx Imaging System (700 nm channel, ex/em 685/720 nm). For CRISPR activation studies a Molecular Devices Spectra Max M5 plate reader was used at 37 °C (bottom read, ex/em 600/660 nm).

#### Fluorescence microscopy

Cells were seeded in 96 well black-wall, clear bottom plates as described above. 10 µM AVX235 was added to cells 1.5 hours prior to addition of probe. Probe was added as described above and cells were incubated for 2.5 hours. Medium was then removed and 100 µL of phenol red and FBS free medium (37 °C) was added. 1 µL of HOECHST 333258 nuclear stain was added. Images were acquired on a Leica SP8 imaging system and analyzed with Image J.

#### Animal studies

All animal studies were approved by the Institutional Animal Care and Use Committee at the University of Pennsylvania and all methods were performed in accordance with the relevant guidelines and regulations. Mice were purchased at 4–6 weeks of age. Weight was measured on arrival and monitored biweekly. Mice were euthanized in the event of rapid weight loss.

#### Tumor inoculation

Cancer cells at ~80% confluency were trypsinized, washed twice in PBS, diluted to 2 × 10^7^ cells/mL, and placed on ice. To this suspension, an equal volume of 4 °C Matrigel (Corning, Cat. # CB-40234A) was added. Prior to inoculation, mice were anesthetized with isoflurane (2–4%, 1 L/min O_2_) and the area of injection was sterilized with 70% isopropanol. The suspension was mixed thoroughly and drawn into a 1 mL syringe. Air bubbles were removed through tapping and the syringe was depressed until a continuous stream of media was released. Tumor xenografts were generated through injection into the right or left lower flank or superior shoulder flank. Orthotopic tumors were generated through injection into the mammary fat pad inferior to the lower nipple of the mouse. 1 × 10^6^ cells were injected per tumor.

#### Optical imaging

Fluorescence was acquired on either the PerkinElmer IVIS Spectrum using a 640 nm excitation filter and a 680 nm emission filter (Bin: (M)8, FOV: 22.3 cm^2^, f2, 3s exposure) or a LI-COR Pearl Impulse using the 700 nm fluorescence channel (ex/em: 685/720 nm). IVIS Spectrum mages were analyzed with LivingImage 4.4 and fluorescence is reported as Average Radiant Efficiency ([p/sec/cm^2^/sr]/[µW/cm^2^]). LI-COR Pearl images were analyzed with Image Studio Lite and fluorescence is reported in relative fluorescence units.

#### *Ex Vivo* tumor painting

Untreated mice bearing orthotopic tumor xenografts were euthanized and tumor tissue was resected. Tissue was washed 1x in PBS and sliced using a razor to approximately 1 mm sections, which were then weighed and placed in 37 °C DMEM with 10% serum and cultured for 1 hour. The medium was replaced with phenol red free and serum free DMEM. 100 µL of liposomes containing 20 nmol probe was then pipetted evenly on top of the tissue and the tumors were imaged in the IVIS Spectrum.

#### Intraoperative activation

Mice were inoculated with 4175-Luc+ or MCF-7 cells in the lower mammary fat pad to generate orthotopic tumors and in the upper shoulder to generate tumor xenografts. Following formation of palpable tumors, DDAO arachidonate was injected i.p. directly under orthotopic tumors growing in the lower mammary fat pad adjacent to the peritoneal cavity. Following 1 hour, mice were euthanized, the peritoneal cavity was exposed and fluorescence was acquired (ex/em: 640/680 nm). Tumors and fat pads were then removed by dissection, washed 1x with PBS, and imaged for fluorescence (ex/em: 640/680).

#### Whole body activation

Mice were anesthetized with isoflurane and 40 nmol of DDAO arachidonate or palmitate was injected i.v. Fluorescence was acquired (ex/em: 640/680 nm) at indicated time-points in the PerkinElmer IVIS Spectrum. After completion of the study mice were euthanized, tumors and organs were removed via dissection, washed 1x in PBS, and evaluated for fluorescence (ex/em: 640/680 nm).

#### Protein immunoblot

Cells were seeded in 10 cm dishes at 2.5 × 10^5^ cells/mL in 12 mL of medium. Following a 24 hour incubation, the cells were washed twice with 4 °C PBS and scraped on ice in radio immunoprecipitation assay buffer containing cOmplete mini EDTA-free protease inhibitor cocktail (Roche). Cells were vortexed and subjected to three cycles of freeze-thaw in liquid nitrogen with thawing at 37 °C. Cells were sonicated in a water bath sonicator at 4 °C for 5 minutes, and left on ice for 15 minutes. Cellular debris was pelleted by centrifugation (13,000 rpm, 10 min, 4 °C). Supernatant was removed and protein concentration was measured with the bicinchonic acid (BCA) protein assay kit (Pierce). Following equalization of protein concentration throughout samples, the lysate was diluted, 1:1 in Laemmli loading buffer (5% 2-mercaptoethanol), and heated to 95 °C for 5 minutes. 30 µL of approximately 25 µg protein were loaded per well. Samples were separated on a 10% SDS-PAGE gel and transferred to 0.45 µm pore nitrocellulose membrane (Invitrogen). The membrane was blocked in Odyssey blocking buffer (LI-COR Biosciences), incubated with primary and secondary antibodies, and visualized using the LI-COR Odyssey CLx Imaging System. Images were quantified with Image Studio Lite.

#### CRISPR genetic knockdown

4175-Luc+ cells were seeded at 1.5 × 10^5^ cells in 6 well plates in antibiotic free DMEM. Following 24 hours, 2 µg CRISPR cPLA2α or scrambled Plasmid (Santa Cruz Biotechnology) was diluted in 150 µL Plasmid Transfection medium (Santa Cruz Biotechnology). The solution was added to 150 µL Plasmid Transfection medium containing 10 µL UltraCruz^®^ Transfection Reagent. Samples were left to incubate for 20 minutes then added dropwise to cells. The cells were incubated for 72 hours. Successfully transfected cells were sorted for GFP fluorescence on a BD FACSJAZZ. Knockout of cPLA2α was analyzed using Western Blot, and cell stocks were frozen in liquid nitrogen. Complete knockout of cPLA2α was not achieved, but a knockdown was observed. cPLA2α knockdown cells were expanded in T25 flasks and used for 5 passages before being discarded.

#### Statistical analysis

All data are reported as mean ± standard deviation, unless otherwise noted. Data are listed as an average of three experimental results (n = 3), unless otherwise noted. P values were reported using a two-tailed Student’s t-test and P values of < 0.05 were considered significant.

#### Datasets

The datasets generated during and/or analyzed during the current study are available from the corresponding author on reasonable request.

## Supplementary information

Supplementary Information
