# Supplementary material for: Detection and Differentiation of Breast Cancer Sub-Types using a cPLA2α Activatable Fluorophore
Source: Sci Rep. 2019 Apr 16;9:6122. doi: 10.1038/s41598-019-41626-y (PMC6467920; doi:10.1038/s41598-019-41626-y)
Supplement: Supplementary file 1 — Supplementary Information [file 41598_2019_41626_MOESM1_ESM.docx]

**SUPPORTING INFORMATION**

**Detection and Differentiation of Breast Cancer Sub-Types
using a cPLA2α Activatable Fluorophore**

Chiorazzo, Michael G.^a^, Tunset, Hanna Maja^b^, Popov, Anatoliy V.^a^, Johansen, Berit^c,d^,

Moestue, Siver^b,e^, Delikatny, E. James^a, *^

^a^ Department of Radiology, Perelman School of Medicine, University of Pennsylvania, Philadelphia, Pennsylvania 19104, United States

^b^ Department of Circulation and Medical Imaging, Norwegian University of Science and Technology, Trondheim 7491, Norway

^c^ Department of Biology, Norwegian University of Science and Technology, Trondheim 7491, Norway

^d^ Avexxin AS, Department of Biology, Norwegian University of Science and Technology, N-7491 Trondheim, Norway

^e^ Department of Laboratory Medicine, Children’s and Women’s Health, NTNU, The Norwegian University of Science and Technology, Trondheim 7489, Norway

* Corresponding Author:

James Delikatny, Ph.D.

317 Anatomy Chemistry Building,

3620 Hamilton Walk,

Philadelphia, PA 19104

Phone: +1(215) 898-3105; E-mail: delikatn@mail.med.upenn.edu


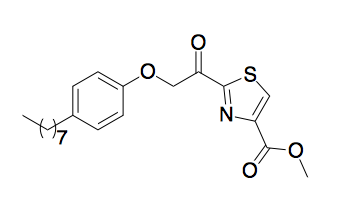


**Figure S1. Structure of the cPLA2α inhibitor, AVX235.**


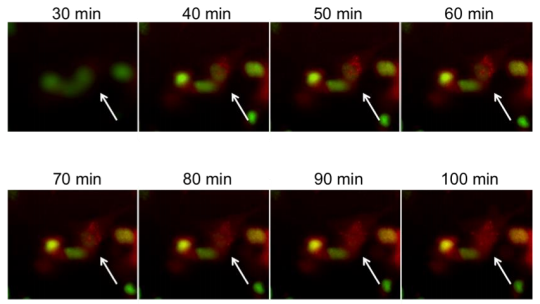


**Figure S2. DDAO arachidonate activation with time.** Wide field fluorescence microscopy was used to visualize changes in subcellular probe activation in 4175-Luc+ cells with time. DDAO fluorescence is shown in red, nuclear stain (Syto 9) is shown in green. Fluorescence is initially seen in small bright vesicles surrounding the nucleus, and then disperses uniformly throughout the cytoplasm with time.


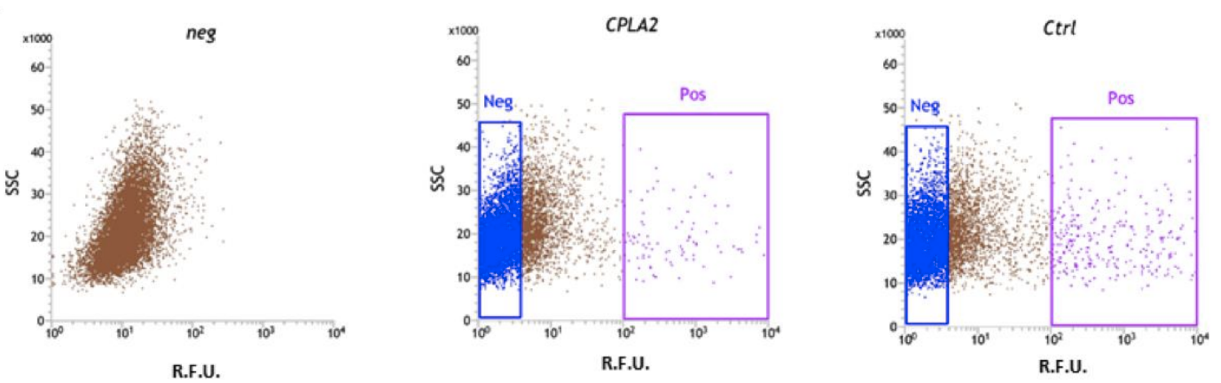


**Figure S3. CRISPR/Cas9 knockout of cPLA2 in 4175-Luc+ Cells.** Following transfection and incubation of 4175-Luc+ cells with CRISPR/Cas9 cPLA2 and scrambled knockout vector, cells were sorted for GFP fluorescence. Non-transfected cells (Neg) were used to define a fluorescence cut-off of 10^2^ R.F.U. for sorting of cPLA2 transfected (CPLA2) and scrambled transfected (Ctrl). Positive cells were isolated, cultured and used for 5 passages as a cPLA2 knockdown line.


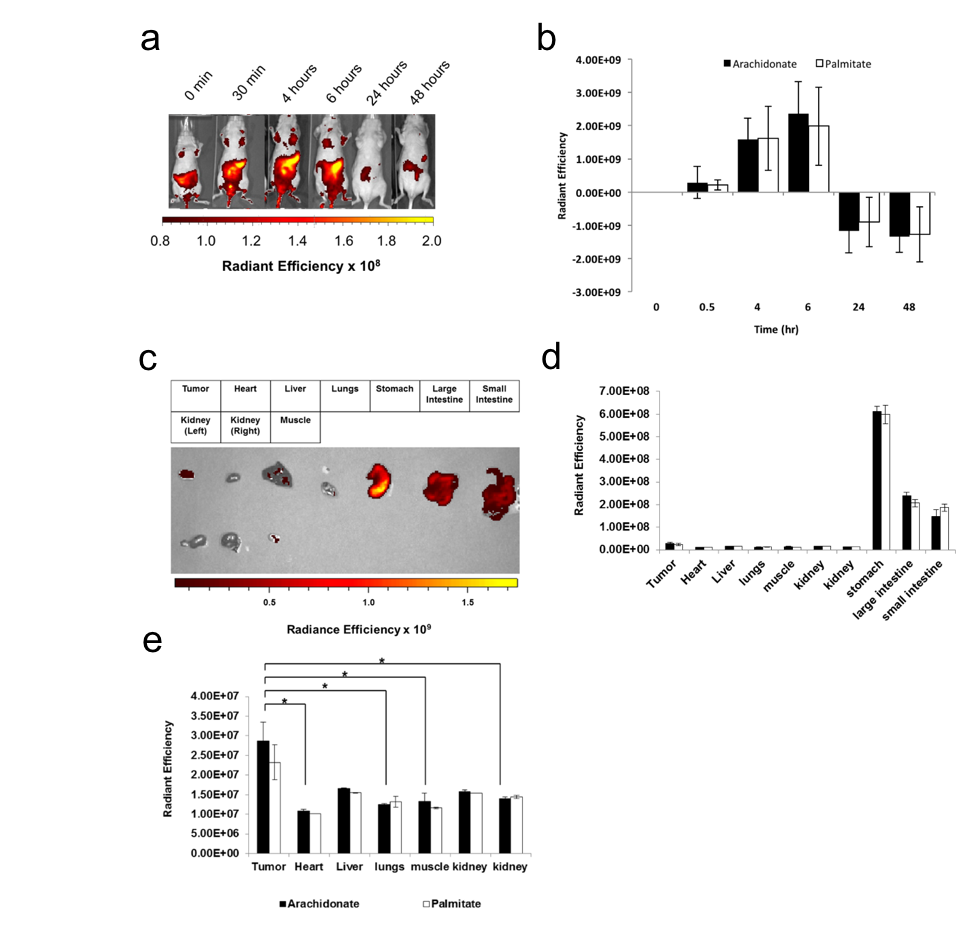


**Figure S4. Whole body fluorescence imaging of DDAO arachidonate and DDAO palmitate.** (A) DDAO arachidonate fluorescence (ex/em: 640/680 nm) in mice following i.v. injection of 40 nmol probe. Ventral images reveal high fluorescence activation occurring in the intestines up to 6 hours and dissipating at 48 hours. (B) Quantification of whole body fluorescence following i.v. injection of 40 nmol DDAO arachidonate or DDAO palmitate. No significant differences between cPLA2 and control probe is seen with time. (C) Ex vivo fluorescence at 48 hours of individual organs in mice treated with 40 nmol DDAO arachidonate through tail vein injection. (D) Quantification of organs shown in C following treatment with either DDAO arachidonate or DDAO palmitate. (E) Quantification of non-digestive tract organs, taken from D.


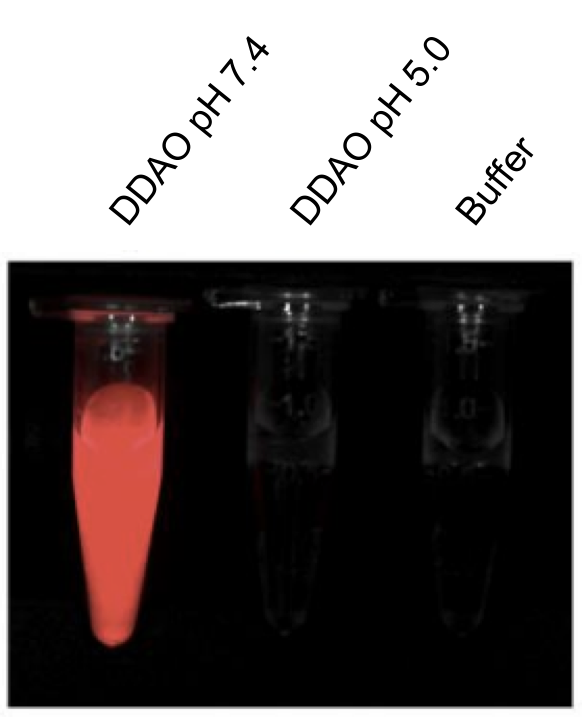


**Figure S5. DDAO fluorescence in the LI-COR Pearl Imaging System.**  DDAO dissolved in pH 7.4, pH 5.0 TRIS buffer in addition to buffer alone. Fluorescence was acquired in the LI-COR Imaging System (700 nm channel). DDAO fluorescence is observed at pH 7.4, but not pH 5.0. At low pH the hydroxyl group in DDAO is protonated, which depletes fluorescence, mimicking the caged fluorescence seen with esterification.


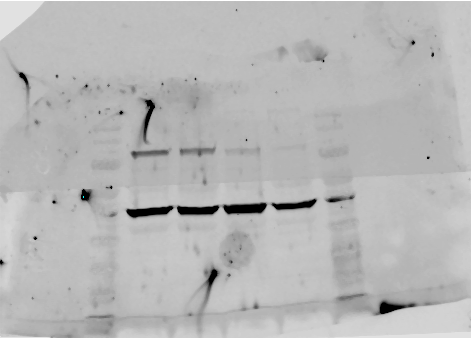


**Figure S6. DDAO arachidonate activation in cell lines varying in cPLA2α expression.** Full membrane showing cPLA2 (top) and ß-tubulin (bottom), presented as a cropped image with quantitation in Fig 2. Cell lines from left to right: 4175Luc+, MDA-MB-231, SKBR3, and MCF-7. Outside lanes contain a protein ladder for size determination.
